# Neuroinflammation in CTLA-4 Haploinsufficiency: Case Report of a New Variant with Remarkable Response to Targeted Therapy

**DOI:** 10.3390/ijms26189230

**Published:** 2025-09-21

**Authors:** Letizia Baldini, Lucia Del Vecchio, Sara Cerasi, Anna Fetta, Mattia Moratti, Alessandra Bezzi, Simona Ferrari, Guido Di Dalmazi, Simone Rossi, Francesco Toni, Duccio Maria Cordelli, Marcello Lanari, Francesca Conti

**Affiliations:** 1 Pediatric Unit, IRCCS Azienda Ospedaliero-Universitaria di Bologna, 40138 Bologna, Italyfrancesca.conti27@unibo.it (F.C.); 2 Department of Medical and Surgical Sciences (DIMEC), University of Bologna, 40138 Bologna, Italy; 3 Specialty School of Paediatrics, University of Bologna, 40138 Bologna, Italymattia.moratti@studio.unibo.it (M.M.); 4 Pediatric Hematology and Oncology, IRCCS Azienda Ospedaliero-Universitaria di Bologna, 40138 Bologna, Italy; 5 UOC Neuropsichiatria dell’Età Pediatrica, IRCCS Istituto delle Scienze Neurologiche di Bologna, 40139 Bologna, Italy; 6 Department of Systems Medicine, University of Tor Vergata, 00133 Rome, Italy; 7 Medicina Interna e Reumatologia/Struttura Semplice di Reumatologia Rimini ASL Romagna, 47923 Rimini, Italy; 8 UO Genetica Medica, IRCCS Azienda Ospedaliero-Universitaria di Bologna, 40138 Bologna, Italy; 9 Division of Endocrinology and Diabetes Prevention and Care, IRCCS Azienda Ospedaliero-Universitaria di Bologna, 40138 Bologna, Italy; 10 Neurologia Azienda Ospedaliero-Universitaria, IRCCS Istituto delle Scienze Neurologiche di Bologna, 40124 Bologna, Italy; 11 Programma di Neuroradiologia con Tecniche ad Elevata Complessità, IRCCS Istituto delle Scienze Neurologiche di Bologna, 40124 Bologna, Italy

**Keywords:** neuroinflammation, autoimmunity, inborn errors of immunity, CTLA-4 deficiency, targeted therapy, abatacept

## Abstract

Inborn errors of immunity (IEI) encompass a diverse group of genetic disorders that often present with complex and multifaceted clinical features, including neuroinflammation. CTLA-4 deficiency (CTLA-4d), caused by monoallelic germline mutations in the CTLA4 gene, manifests with autoimmune phenomena, lymphoproliferation, infections, and neurological involvement in up to 30% of patients, with a broad clinical spectrum, ranging from encephalitis to demyelination and lymphocytic infiltration. Imaging typically shows multifocal contrast-enhancing lesions. Early recognition of CTLA-4d is crucial to guide clinical management. Herein, we report the case of a 15-year-old girl presenting with severe multifocal neuroinflammatory lesions, recurrent infections, and systemic granulomatous disease. After extensive infectious and immunological workup, a heterozygous de novo CTLA4 variant c.394G>A_p.Glu132Lys was identified and its pathogenicity confirmed by transendocytosis functional assays. Based on the genetic diagnosis, the patient was started on abatacept, with brilliant clinical and radiological results after dose adjustment. This report describes a new pathogenic variant of the CTLA4 gene and highlights the importance of considering IEIs, such as CTLA-4d, in patients with unexplained severe neuroinflammation. Also, it highlights the efficacy and tolerability of abatacept as a targeted therapy for neuroinflammation in CTLA4-d.

## 1. Introduction

Inborn errors of immunity (IEI) constitute a heterogeneous group of genetic disorders. Advances in genetic technologies have enhanced our understanding of their underlying molecular mechanisms and facilitated the development of targeted therapies capable of modifying disease progression. Monoallelic germline mutations in the Cytotoxic T-lymphocyte antigen-4 (CTLA4) gene result in CTLA-4 deficiency (CTLA-4d), a complex IEI characterized by a broad and multifaceted clinical spectrum, including various autoimmune manifestations, lymphoproliferation, and recurrent infections [1]. Neurological involvement occurs in up to 30% of affected patients. It is typified by multifocal inflammatory lesions in the central nervous system (CNS), which may mimic multiple sclerosis (MS) or present with seizures and encephalopathy [2]. Herein, we describe a patient harboring a novel pathogenic CTLA4 variant who exhibited prominent multifocal neurological symptoms and a remarkable response to targeted therapy with abatacept.

## 2. Case Report

**Figure 1:**
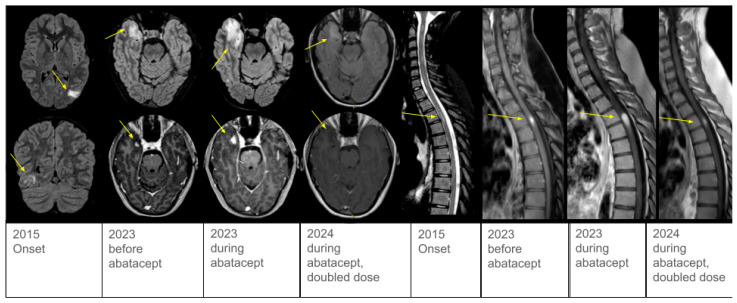
MRI evolution. On the left side: axial FLAIR T2-weighted sequences and post-gadolinium axial 3D IR T1-weighted sequence performed at onset, before abatacept initiation, during standard-dose abatacept therapy, and after dose escalation. On the right side: sagittal cervico-thoracic post-gadolinium 2D T1-weighted images performed at onset, before targeted therapy, during standard-dose abatacept therapy, and after dose escalation. After abatacept initiation, both the right temporal lesion and the spinal lesion worsened, with increased contrast enhancement and perilesional vasogenic edema. All the lesions improved after doubling the abatacept dose.

A 15-year-old girl, with a previous history of headaches and recurrent respiratory infections, presented with new-onset focal epileptic seizures. Her family history was unremarkable. Brain magnetic resonance imaging (MRI) showed multiple T2-hyperintense lesions with contrast enhancement involving the cerebral and cerebellar gray and white matter, consistent with inflammatory lesions (Figure 1). Cerebrospinal fluid (CSF) analysis disclosed elevated white blood cell count and protein levels. Abdominal and lung computed tomography (CT) scans revealed splenomegaly and diffuse lung nodularities, which were confirmed as granulomatous infiltrates on biopsy. Positron-emission tomography (PET) scan displayed heightened metabolic activity in the mediastinal, cerebral, and abdominal regions.

**Figure 2:**
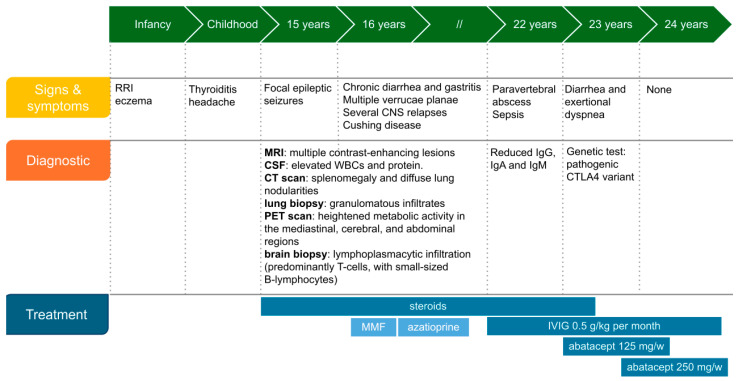
Overview of clinical history and management. Abbreviations: RRI: recurrent respiratory infections, CNS: central nervous system, WBC: white blood cells, MMF: mycophenolate mofetil 600 mg/m^2^ bid; azathioprine 2 mg/kg orally per day; IVIG: intravenous immunoglobulins.

After exclusion of infectious diseases, including negative cytomegalovirus, EBV, HIV, and Mycobacteria tests, a potential systemic inflammatory/immune-mediated disorder was considered. The patient was initiated on intravenous (IV) methylprednisolone (1 mg/kg/day), leading to regression of the cerebral and pulmonary lesions within one month. However, during corticosteroid tapering, the patient experienced a severe relapse; MRI revealed recurrent multiple brain lesions associated with initial compressive hydrocephalus, necessitating a decompressive craniotomy. Histological analysis of the brain biopsy showed lymphoplasmacytic infiltration (T cells and small-sized B-lymphocytes) with extensive perivascular cuffs, without fibrinoid deposits or vascular wall damage. The infiltrates exhibited a granulomatous appearance. A detailed timeline of the medical history is represented in Figure 2.

Over the following years, the patient remained steroid-dependent, experiencing recurrent CNS relapses and new-onset spinal cord involvement upon steroid tapering (Figure 1). Steroid-sparing immunosuppressants—such as azathioprine and mycophenolate—were attempted but discontinued due to inefficacy or gastrointestinal adverse effects, respectively.

### 2.1. Immunologic Workup

At age 22 (seven years after disease onset), she was referred to our Immunology Outpatient clinic. Comprehensive immunological workup showed panhypogammaglobulinemia (IgG 262 mg/dL, IgM 42 mg/dL, IgA 7 mg/dL), lymphopenia (1170 cells/µL), reduced absolute B cells (20 cells/µL, 1.4% of lymphocytes), and decreased percentages of naïve CD4 T cells (10% of CD3+CD4+). Due to the extremely low number of B cells, further characterization of B subsets was not feasible. The T cell response to mitogens showed 85% proliferating cells. After the initiation of abatacept, the B cell count doubled, showing a severe decrease in percentage of memory and switched memory. Complete immunologic workup is shown in Supplementary Table S1. Overall, the patient fulfilled the diagnostic criteria for probable Common Variable Immunodeficiency [3], and immunoglobulin replacement therapy was immediately initiated. Notably, the patient also had diffuse plane warts, mild enteritis, and mild lung involvement.

### 2.2. Genetic Diagnosis and Targeted Therapy

A targeted Next Generation Sequencing (NGS) panel identified a heterozygous de novo variant c.394G>A_p.Glu132Lys in exon 2 of the CTLA4 gene. This missense variant is unreported in gnomAD and has been reported once in ClinVar as a variant of unknown significance (VUS). In silico pathogenicity prediction tools suggested that the variant might be potentially damaging. No other potential pathogenic variants were detected.

Upon identification of the VUS, subcutaneous abatacept was started at a dosage of 125 mg/week. Brain and spine MRI before abatacept initiation are shown in Figure 1. Neurological examination at that time revealed only mild sensory loss in her left arm and trunk.

Six months after initiating targeted therapy, control brain and spine MRI showed worsening lesions (Figure 1), and diarrhea and exertional dyspnea persisted. However, clinical evaluation revealed no signs of spinal cord dysfunction, and somatosensory evoked potentials were within normal range, confirming a clinical–radiological dissociation of disease activity. Consequently, the frequency of abatacept infusions was doubled. Reevaluation with MRI three months later demonstrated improvement and no new lesions (Figure 1). Gastrointestinal and pulmonary symptoms also improved. At the last follow-up, the patient reported good tolerability, improved quality of life, and perceived well-controlled disease. Last immunological tests are reported in Supplementary Table S1.

Meanwhile, CTLA-4 function was assessed by measuring the trans-endocytosis of CD80 via flow cytometry, as previously described [4]. The test was kindly performed by the University of Freiburg. Trans-endocytosis was assessed in CD4+ cells from the patient and two healthy donors, one provided by the laboratory (internal) and one sent with the patient’s sample (traveler control), to exclude falsely positive results due to non-optimal conditions during transport. Results (see Supplementary Figure S1) demonstrated a reduced percentage of trans-endocytosis in the patient’s Treg cells (52.1%) compared to the traveler (77.4%) and internal control (77.2%). Additionally, the percentage of intracellular CTLA-4 high expression in activated Treg cells was markedly reduced (1.57% vs. 41.9%), confirming the pathogenicity of the variant [4,5].

## 3. Discussion

CTLA4d is a rare autosomal dominant IEI with incomplete penetrance characterized by reduced expression of CTLA4, an inhibitory receptor expressed on activated and regulatory T lymphocytes. CTLA4 outcompetes CD28 for binding to CD80/CD86, thereby preventing uncontrolled T cell hyperactivation and lymphoproliferation [6,7]. This condition can manifest with hypogammaglobulinemia associated with recurrent respiratory infections, autoimmune features, lymphoproliferation, and multisystemic lymphoid or granulomatous infiltrations [2].

In our patient, the c.394G>A (p.Glu132Lys) variant was identified through targeted sequencing of genes involved in immunodeficiencies using NGS technology. This missense variant results in an amino acid substitution within exon 2. To date, 30 pathogenic and 11 likely pathogenic variants of the CTLA-4 gene have been reported in association with autoimmune lymphoproliferative syndrome, according to ClinVar [8]. Most of these variants are located in exon 2 [1,9]; mutations in this exon impair the binding of CD80/CD86, potentially explaining the remarkable clinical response to abatacept observed in our patient. Furthermore, CTLA4 mutations are known to affect the Treg cells’ capacity to control CD80 and CD86 levels [4], consistent with the functional assay result [5]. Based on these findings, the novel CTLA-4 mutation identified in our patient was confirmed to be pathogenic.

As far as immunologic phenotype is concerned (see Supplementary Table S1), our patient had an absolute and relative severe B lymphopenia. This observation is common in patients with CTLA-4d (up to 41%) and initially prevented us from further characterizing B cell subsets. After the initiation of abatacept, we were able to detect a severe decrease in percentage of memory and switched memory B cells, with a relative increase in naïve B cells, consistent with previous observations and concurrent hypogammaglobulinemia. Conversely, T cell counts were within the normal range, with reduced naïve pool, in accordance with the biggest published CTLA4-d cohort [9].

The neurological spectrum of CTLA4d is broad, encompassing autoimmune encephalitis, perivascular lymphocytic infiltration, inflammatory demyelinating disorders, optic neuritis, CNS atrophy, and lymphoproliferative disorders with CNS infiltration [10]. Previous series reported neurological involvement in about 21–30% of CTLA4 mutation carriers [2,7,11]. Consistent with previous reports in the literature, our patient’s first symptom was recurrent headache, followed by seizures [6,7,10,11,12,13,14]. Other reported neurologic symptoms include sensory–motor deficits, visual hallucinations, vision disturbances, sphincter dysfunction, early cognitive decline, and visual impairment [6,10,12,15]. Seizures in CTLA4 deficiency often result from encephalic inflammatory infiltration [10], although some reported cases presented with tonic–clonic seizures for which clinical and radiologic investigation could not identify an underlying cause [9]. The median age at the discovery of CNS inflammation is reported to be 18–18.8 years [7,11]. In our case, seizures were the first symptom, revealing early-onset encephalic lesions and multisystem involvement. Usually, neuroinflammation appears 9.2 to 12 years after initial systemic signs (median) [7,11]. Based on our experience, we recommend considering a potential CTLA4-d in unexplained neuroinflammation, as CNS can rarely be the presenting manifestation.

In terms of neuroimaging, the most common radiological findings include multifocal intraparenchymal T2-hyperintense and contrast-enhancing lesions [2]. Strikingly, the severity of the lesions did not correspond to the symptoms reported by our patient, nor to the neurological examination findings. Notably, our patient showed an important improvement in neurological symptoms with a standard dose of abatacept, and pneumologic and gastrointestinal symptoms also resolved. Nonetheless, MRI showed a worsening enlarged thoracic spinal cord lesion with radiological prominent swelling and contrast enhancement (Figure 1), resulting in no neurologic impairment nor evoked potential abnormalities. Although a clinico-radiological dissociation has already been described in CTLA4-d encephalic lesions, large thoracic lesions are usually symptomatic [7]. Our case highlights that large medullary lesions can also be asymptomatic, so we recommend to always include spinal MRI as part of follow-up, regardless of clinical manifestations. To date, and except for the Schindler’s cohort, the description of neuroinflammation in patients with CTLA4-d mainly consists of case reports, detailing the diagnosis and treatment with a limited follow-up; conversely, due to the diagnostic delay, the follow-up period of our patient lasted more than ten years. During this time, some lesions grew up to cause compressive hydrocephalus, others—after an initial phase of growth—tended to spontaneously decrease in size, with a characteristic ‘waxing and waning’ pattern. Furthermore, new lesions kept on appearing over the years, until targeted therapy was successfully escalated. Our case confirms a previous observation that CNS in CTLA4-d can frequently relapse and details the natural course of the disease over a 10-year observation period, proving that neuroinflammation severity is quite unrelated to systemic symptoms [7]. Based on our experience, the neurological follow-up in these patients can be really challenging, so we suggest relying on serial imaging rather than on clinical examination.

As the CSF analysis often presents nonspecific findings, a biopsy is usually necessary. When performed, polyclonal lymphocytic infiltrate with predominance of T cells (mainly CD4+) is the most common feature, together with perivascular inflammation. Also, demyelinating processes can be observed, as well as granuloma formation, like in our patient [6,7,11,13,14,15]. These lesions are dynamic over time, can be found both in the brain and in the spinal cord, and are presumably caused by T cell hyperactivation. Brain resident regulatory Treg seems to play a crucial role in controlling CNS inflammation and preventing autoimmunity, as demonstrated in patients with multiple sclerosis [16]. The inhibitory pathways that lie downstream of CTLA4 activation are decisively engaged in Treg functionality and the maintenance of CNS immune homeostasis. Accordingly, neuroinflammation in CTLA4d might be compared to some frequent adverse effects of CTLA4-inhibitor treatment (e.g., ipilimumab) [17]. Intriguingly, despite a common dysfunctional pathway, neurological toxicities associated with CTLA4 inhibitors more commonly involve the peripheral nervous system, while neurological involvement in CTLA4d is exclusively limited to the CNS [18].

High-dose corticosteroids are the first-line therapy for neuroinflammation in CTLA-4d. In a global cohort of 123 patients, including 41 with neuroinflammation, Egg et al. reported a symptomatic clinical response in 65% of patients treated with various doses of corticosteroids [2]. Nonetheless, most patients show persistent or relapsing lesions during tapering. Abatacept and sirolimus may be initiated for optimizing treatment [2]. Abatacept, a fusion protein that includes CTLA4 and the Fc part of IgG1, has the potential to bind to CD80/86 on the antigen-presenting cells, blocking CD28 binding and hampering T cell activation [19]. Our patient was steroid-dependent. Therefore, following the diagnosis, we optimized therapy by introducing abatacept at the standard dose of 125 mg/week [2] while tapering steroids. Since the patient showed a good clinical response, but limited radiological improvement, and in light of previous reports describing the use of higher dosages (187.5–350 mg/week) [11,20], we decided to escalate the dose to 250 mg/week. This adjustment, guided both by the literature data and the individualized clinical response, resulted in a marked clinical–radiological improvement with good tolerability. Similar positive responses were reported by other authors [2,10,11,14,15,16,17,18,19,20,21,22]. Regarding sirolimus, although widely recommended to control immune dysregulation in CTLA-4d [2,22], we identified only one report specifically describing its use to treat neuroinflammation in two patients, both showing a good response [6].

## 4. Conclusions

In cases of severe and refractory neuroinflammatory presentations, it is essential to consider IEIs as an underlying cause. CNS involvement can rarely be the presenting manifestation. Timely genetic diagnosis not only improves understanding of the pathogenesis but, more importantly enables the initiation of targeted therapies—such as abatacept in CTLA-4 deficiency—with a significant positive impact on patients’ quality of life. Our findings expand current knowledge by showing that CTLA4-d may present with large, asymptomatic spinal cord lesions, highlighting the need for systematic CNS monitoring. Moreover, the long-term follow-up documents the dynamic evolution of neuroinflammation, with waxing-and-waning lesions, underscoring the importance of early targeted therapy. Last but not least, this case therefore contributes to the current understanding of abatacept use in CTLA4-d, suggesting that dose escalation beyond the standard regimen may be required in selected patients to achieve optimal disease control, and highlighting the need for further studies to define tailored dosing strategies.

## Data Availability

The principal authors take full responsibility for the data, the analyses, and interpretation; have full access to all of the data; and have the right to publish any data, separate and apart from the guidance of any sponsor. Data are available upon request.

## Supplementary Materials

The supporting information can be downloaded at: https://www.mdpi.com/article/10.3390/ijms26189230/s1.

## Abbreviations

The following abbreviations are used in this manuscript:

CNS	central nervous system
CSF	cerebrospinal fluid
CT	cerebrospinal fluid
CTLA-4d	CTLA-4 deficiency
IEI	inborn errors of immunity
IV	intravenous
MRI	magnetic resonance imaging
MS	multiple sclerosis
NGS	next generation sequencing
PET	positron-emission tomography
VUS	variant of unknown significance
