# Supplementary material for: Neuroinflammation in CTLA-4 Haploinsufficiency: Case Report of a New Variant with Remarkable Response to Targeted Therapy
Source: Int J Mol Sci. 2025 Sep 21;26(18):9230. doi: 10.3390/ijms26189230 (PMC12470878; doi:10.3390/ijms26189230)
Supplement: Supplementary file 1 [file ijms-26-09230-s001.zip › Supplementary material CTAL4.pdf]

Supplementary Figure S1: CTLA-4 variant validation. Primary human CD4+ T cells were purified from PBMCs by negative selection and activated with CD3/CD28 beads for 16 hours in the presence of Chinese hamster ovary (CHO) cells expressing CD80-mScarlet. After incubation, cells were harvested and gated on CD4+CD45RO+FOXP3+ (Tregs) and analyzed for CD80-mScarlet uptake.Transendocytosis was assessed in CD4+ cells from the patient and two healthy donors: one provided by the laboratory (internal) and one sent with the patient's sample (traveler control). **A:** Results of transendocytosis by flow cytometry. **B:** Results of CTLA-4 expression by flow cytometry.

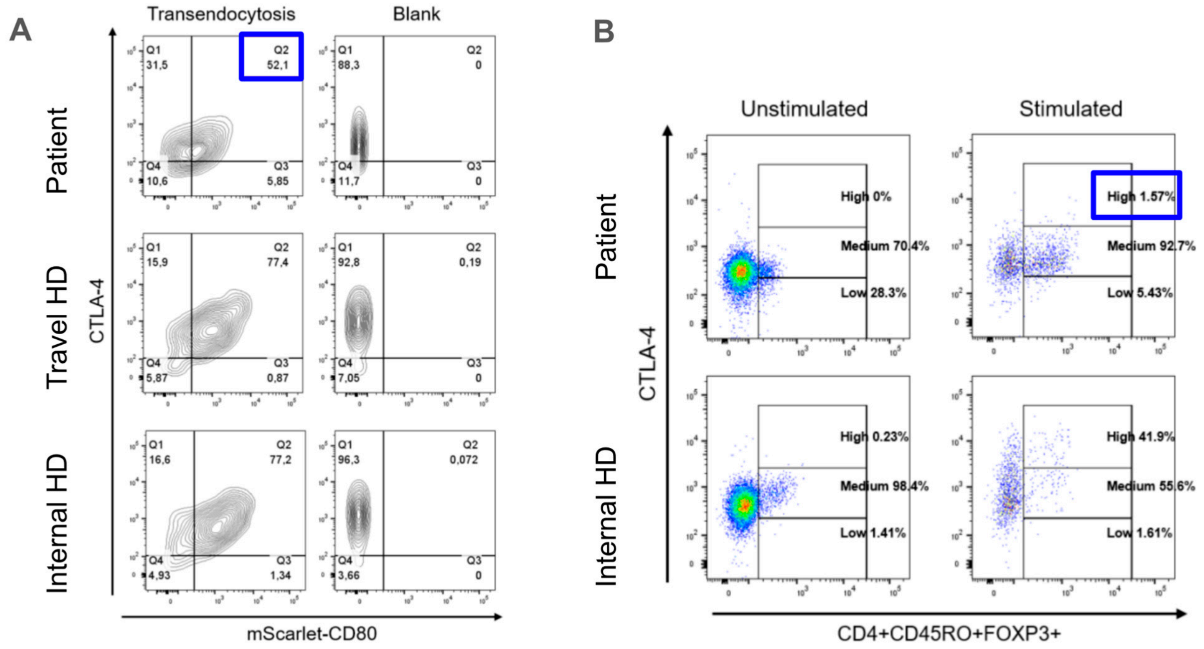

Supplementary Table S1: Immunological exams prior and post Abatacept. The first workup was made during steroid therapy. Last workup was made during abatacept 250mg/week.
IRT, Immunoglobulin replacement therapy.
References: Comans-Bitter WM,et al. Immunophenotyping of blood lymphocytes in childhood. Reference values for lymphocyte subpopulations. J Pediatr. 1997 Mar;130(3):388-93.

	**November 2022**	**September 2025**
White blood cells [cells/μL]	5409	7100
Hemoglobin [g/dL]	13,9	14,7
Platelets [cells/μL]	154.000	150.000
Neutrophils [cells/μL]	3690	5480
Lymphocytes [cells/μL]	1170	1130
CD3+ % [cells/μL]<br>(reference values)	90% [1050]<br>(55-83%) [700-2100]	75% [830]<br>(55-83%) [700-2100]
CD3+/α+β+ [% of CD3+]	99,0	99,0
CD3+/γ+δ [% of CD3+]	1,0	1,0
CD3+/α+β+CD4-CD8- [% of CD3+]	0,9	0,7
CD3+CD4 % [cells/μL]<br>(reference values)	71,0% [830]<br>(28-57%)[300-1400]	50% [565]<br>(28-57%) [300-1400]
CD4+CD45 RA+ Naïve [% of CD4+]	10,0	17,3
CD4+CD45 RA-CCR7+ Central memory [% of CD4+]	65,0	65,4
CD4+CD45 RA-CCR7- Effector memory [% of CD4+]	23,0	16,3
CD4+CD45 RA+CCR7- Terminal effector memory [% of CD4+]	1,0	<0,01
CD3+CD8 % [cells/μL]<br>(reference values)	18,0 [210]<br>(10-39%) [200-900]	20,0 [227]<br>(10-39%) [200-900]
CD8+CD45 RA+ Naïve [% of CD8+]	35,0	30,6
CD8+CD45 RA-CCR7+ Central memory [% of CD8+]	30,0	17.0
CD8+CD45 RA-CCR7- (Effector memory) [% of CD8+]	22,0	18.0
CD8+CD45 RA+CCR7- (Late effector) [% of CD8+]	14,0	28.1
CD56+16+CD3- [cells/μL]<br>(reference values)	8 [93]<br>(7-31%) [90-600]	20 [222]<br>(7-31%) [90-600]
CD19 % [cells/μL]<br>(reference values)	1,4 [20]<br>(6-19%) [100-500]	3,4 [40]<br>(6-19%) [100-500]
CD19+IgD+CD27- B naïve [% of CD19]	NA	88
CD19+IgD+CD27+ B memory [% of CD19]	NA	2,4
CD19+IgD-CD27+ switched B memory [% of CD19]	NA	0,8
CD19+CD21+CD38- B CD21+low [% of CD19]	NA	6,3
CD19+IgM++CD38++ B transitional [% of CD19]	NA	14,3
CD19+IgM-+CD38++ B plasmablast [% of CD19]	NA	0,6
IgM [mg/dL]	42	173
IgA [mg/dL]	7	8
IgG [mg/dL]	262 (before IRT)	1160 (during IRT)
T-cell response to mitogens (antiCD3 antiCD28 + recombinant IL-2), %	85	
